# Cerebrovascular reactivity among native-raised high altitude residents: an fMRI study

**DOI:** 10.1186/1471-2202-12-94

**Published:** 2011-09-26

**Authors:** Xiaodan Yan, Jiaxing Zhang, Qiyong Gong, Xuchu Weng

**Affiliations:** 1 Laboratory for Higher Brain Function, Institute of Psychology, Chinese Academy of Sciences, Beijing, China; 2 Department of Physiology, Medical College of Xiamen University, Xiamen, China; 3 Cognitive Science Department, Rensselaer Polytechnic Institute, Troy, NY, USA; 4 Center for Neural Science, New York University, New York, NY, USA; 5 Huaxi Magnetic Resonance Research Center, West China Hospital, Sichuan University, Chengdu, China

## Abstract

**Background:**

The impact of long term residence on high altitude (HA) on human brain has raised concern among researchers in recent years. This study investigated the cerebrovascular reactivity among native-born high altitude (HA) residents as compared to native sea level (SL) residents. The two groups were matched on the ancestral line, ages, gender ratios, and education levels. A visual cue guided maximum inspiration task with brief breath holding was performed by all the subjects while Blood-Oxygenation-Level-Dependent (BOLD) functional Magnetic Resonance Imaging (fMRI) data were acquired from them.

**Results:**

Compared to SL controls, the HA group showed generally decreased cerebrovascular reactivity and longer delay in hemodynamic response. Clusters showing significant differences in the former aspect were located at the bilateral primary motor cortex, the right somatosensory association cortex, the right thalamus and the right caudate, the bilateral precuneus, the right cingulate gyrus and the right posterior cingulate cortex, as well as the left fusiform gyrus and the right lingual cortex; clusters showing significant differences in the latter aspect were located at the precuneus, the insula, the superior frontal and temporal gyrus, the somatosensory cortex (the postcentral gyrus) and the cerebellar tonsil. Inspiratory reserve volume (IRV), which is an important aspect of pulmonary function, demonstrated significant correlation with the amount of BOLD signal change in multiple brain regions, particularly at the bilateral insula among the HA group.

**Conclusions:**

Native-born HA residents generally showed reduced cerebrovascular reactivity as demonstrated in the hemodynamic response during a visual cue guided maximum inspiration task conducted with BOLD-fMRI. This effect was particularly manifested among brain regions that are typically involved in cerebral modulation of respiration.

## Background

In recent years, there have been an increasing population living at high altitude (HA) due to immigration, work duty or tourism [1,2]. How do the environmental challenges at HA impact brain development and brain function, as well as how the brain adapts to HA have raised research concern in recent years, especially with the assistance of neuroimaging research techniques. A PET study on Quechuas, who are indigenous residents at the Andes mountain, suggested that cerebral hypometabolism might have been a defense mechanism against chronic hypoxia [3]. This effect was also replicated on 6 marine soldiers who underwent a 63-day training at HA [4]. Another study with Blood Oxygenation Level Dependent (BOLD) functional magnetic resonance imaging (fMRI) demonstrated decrease in hemodynamic response magnitude associated with short term HA adaptation [5].

Most of previous neuroimaging studies were conducted either on individuals with short-term exposure such as mountain climbers or indigenous HA populations such as Sherpa, Quechuas, Peruvians etc. Neither of these populations are very ideal for studying long term cerebral adaptation, because mountain climbers did not go through long term HA exposure to allow for maximum adaptation; whereas indigenous residents have gone through hundreds years of natural selection, thus active physiological adaptation are confounded with genetic factors (e.g., recently genes were identified on Sherpa for their unique hemoglobin phenotype [6,7]). Therefore, recently our research group has focused on the children of immigrant residents. The direct families of our subjects migrated to HA only 1~3 generations ago, and our subjects were born at HA and grew up at HA until early adulthood (18~24 years old). This population provides a special opportunity to study cerebral adaptation to HA. Previously our research group published several MRI studies on these native born HA residents, in comparison with an age- and ethnic- matched control group that were native sea level (SL) residents [8-11]. We found significantly reduced regional gray matter volumes [8], as well as reduced cerebral responsiveness to gustatory stimulation [9] and during cognitive activity [10]. In the studies with BOLD-fMRI, however, we have been concerned with a fundamental issue, which is the cerebrovascular reactivity (CVR). Because BOLD signal originates from the blood flow and blood oxygenation at microvessals [12,13], differences in CVR properties can influence the spatial and temporal pattern of BOLD signal [14,15]. Therefore we hoped to conduct an experiment to particularly investigate CVR.

In fMRI studies, hypercapnia has been used as a method for demonstrating CVR in the BOLD signal obtained from different populations. CO_2 _inhalation is a straightforward way for inducing hypercapnia [16-19], but it can be invasive. In recent years, researchers have developed tasks with instructed short periods of breath holding as alternative methods, because of the simplicity and non-invasiveness of these methods [14,15,20,21]. There was an influential BOLD fMRI study which suggested the equivalence of breath holding and CO_2 _inhalation in evaluating CVR [22]. There are also studies that suggested the BOLD response to breath holding of short durations were robust [17,20]. In recent years the duration of breath holding in experiments has become shorter and shorter, from 40s [20], to 18s [15], to 2-3s [14,23]. A visual cue guided maximum inspiration task was compared to CO_2 _inhalation, and it was suggested that the brief breath holding (2s) in this task could still induce hypercapnia and it had equivalent effect for observing CVR as CO_2 _inhalation [14]. Experiments also demonstrated that visual cues for instructing voluntary respiration including brief periods of breath holding (2~3 s) could help to control the inspiration level and reduce the variability in BOLD signal [23]. Therefore, considering the reliability and sensitivity of such task, in our experiment we adapted a paradigm of visual cue guided maximum inspiration with brief breath holding.

Another consideration for utilizing this experiment paradigm is our interest in the cerebral modulation mechanism of respiration among the HA residents. "Short-of-breath" is a symptom that new comers to HA frequently complain, but this symptom usually disappears with short term HA adaptation and is absent among long term HA residents [19]. Besides adaptation in peripheral physiology, the cerebral modulation mechanisms should also have significant contribution. Previously researchers have studied adaptation in peripheral physiology [2,24], but cerebral adaptation mechanism are not yet well understood. Our previous study found reduced gray matter volumes at the bilateral insular cortex and the motor cortex [8]. Since these regions are known to be important for respiratory muscle control [25] or respiratory inhibition [26], we suspected that the reduced gray matter volumes at these regions could lead to reduced cerebral activity at these regions, thus helped to reduce hyperventilation among HA residents. This is another reason for us to adapt this experiment paradigm with visual cue guided voluntary respiration.

## Results

### Spirometry results

Spirometry demonstrated that the two groups were similar on the majority of pulmonary function tests (Table 1). Male subjects showed mild group difference on the inspiratory reserve volume (IRV, p < 0.05), with the HA group having a smaller average value. Female subjects did not show difference on any measurements.

**Table 1:** Results of pulmonary function testing

	SL				HA			
	Male		Female		Male		Female	
	Mean	SD	Mean	SD	Mean	SD	Mean	SD
VC	3.72	0.30	2.78	0.34	2.97	1.64	3.19	0.51
IRV	2.06*	0.66	1.09	0.34	1.12*	0.69	1.20	0.38
ERV	1.47	0.44	1.23	0.35	1.38	0.75	1.31	0.33
TV	1.42	0.26	1.41	0.36	2.03	0.65	1.24	0.41
IC	2.25	0.50	1.56	0.34	1.59	1.04	1.88	0.39
MVV	141.29	29.95	97.84	14.09	143.64	29.92	104.04	28.67
RR	16.58	5.12	18.12	6.45	19.61	7.41	19.58	3.31
FVC	3.95	0.37	2.99	0.43	4.01	1.21	3.23	0.47
FEV1	3.36	0.44	2.74	0.27	3.62	0.97	2.37	0.99
MMEF	4.30	1.44	3.69	0.66	4.63	1.10	3.01	1.67
PEFR	8.04	1.64	5.92	0.66	7.22	1.87	4.73	2.37
FEF25	7.62	1.56	5.80	0.70	6.87	2.23	4.53	2.41
FEF50	4.89	1.66	4.17	0.86	4.93	1.37	3.44	1.66
FEF75	2.34	0.96	2.20	0.70	3.27	0.53	1.98	0.74
PEFR/H	4.72	0.95	3.74	0.40	4.11	1.00	2.88	1.43
FEF25/H	4.46	0.88	3.67	0.43	3.90	1.22	2.75	1.46
FEF50/H	2.86	0.94	2.63	0.53	2.80	0.72	2.10	1.01
FEF75/H	1.37	0.54	1.39	0.43	1.87	0.26	1.20	0.43

Abbreviations: VC, vital capacity; IRV, inspiratory reserve volume; ERV, expiratory reserve volume; TV, tide volume; IC, inspiratory capacity; MVV, maximum voluntary ventilation; RR, respiratory rate; FVC, forced vital capacity; FEV1, forced expired volume in one second; MMEF, mean mid-expiratory flow; PEFR, peak expiratory flow rate; FEF, forced expiratory flow, which was taken respectively 25%, 50% and 75% of vital capacity. PEER/H, FEF25/H, FEF50/H, FEF75/H, body height standardized values of corresponding measurements. *: *p *< 0.05 in two sample *t*-test between groups.

### Cerebrovascular reactivity (CVR)

**Figure 1:**
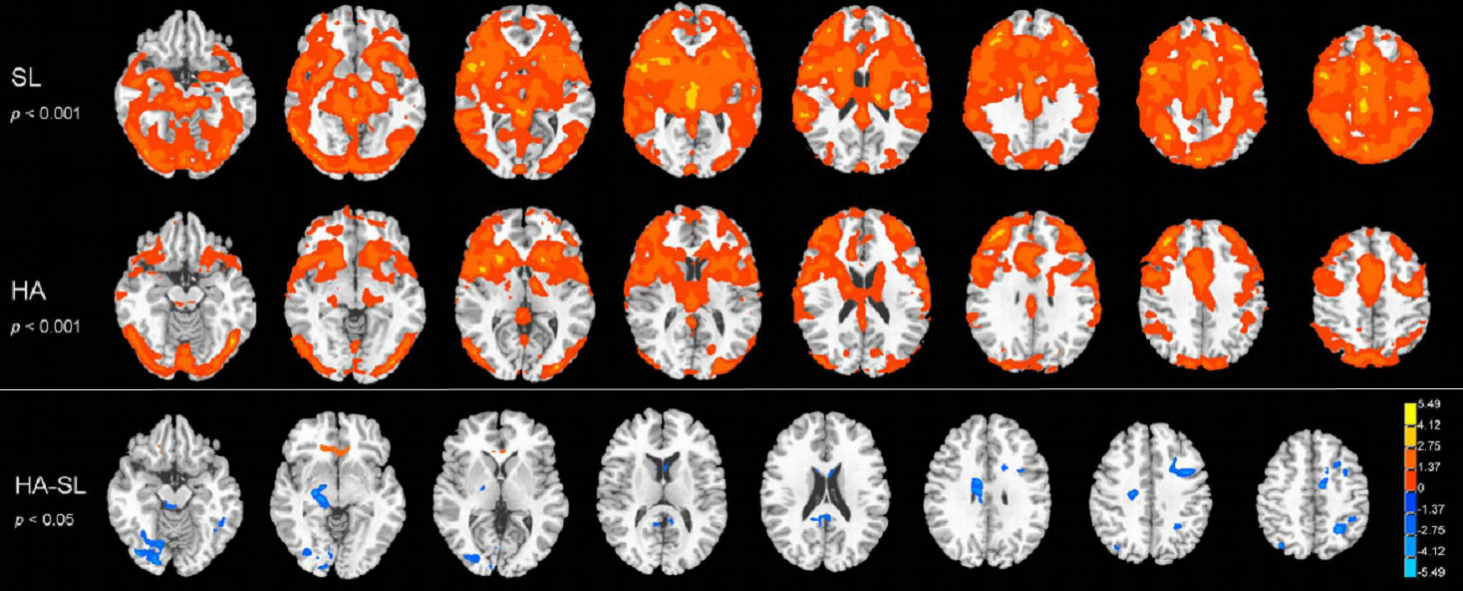
**CVR in both groups as well as group comparison**. The HA group generally showed decreased CVR. Clusters showing significant group differences are displayed in the third row with blue color indicates decreased cereborvascular reactivity among the HA group and red color indicates increase.

**Figure 2:**
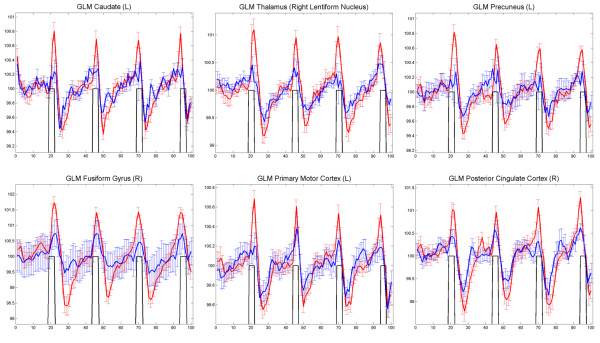
**The average BOLD signal extracted from clusters that demonstrated significantly decreased CVR**. The red lines represent the signal from the SL group and the blue lines represent the signal from the HA group; the black lines represent the task reference; the periods when the black lines are absent are resting periods without performing the cued maximum inspiration task. Error bars are plotted on the average signal of each group at each time point, representing cross-subject variance measured in SD.

CVR was estimated via fitting the BOLD signal of each individual with general linear model (GLM) in a voxel-by-voxel manner. The HA group generally showed lower CVR than the SL group (Figure 1), especially at the primary motor and visual cortex, the somatosensory cortex, the precuneus and the posterior cingulate cortex, the thalamus and the caudate, together with increased CVR at the anterior cingulate cortex (Figure 1, Table 2). The clusters that showed significant group differences were outlined to be used as masks. Then the average of the preprocessed signal was extracted from these masks on each individual subjects and averaged within groups. As demonstrated in Figure 2, the BOLD response signal had smaller amplitudes of change among the HA group (the blue lines) compared to the SL group (the red lines) in the above-mentioned regions that showed decreased CVR. The HA group also had more, if not at a comparable level, cross-individual variability in BOLD signal (the standard error bars plotted on the time series), which is particularly evident at the right fusiform gyrus.

**Table 2:** Detailed information of the clusters showing significant group differences.

Measurements	Area	Volume (mm3)	BA		Talairach (peak)			*t*-value
					x	y	z	
GLM	Primary motor cortex	6854	6	L	21.7	3.9	54.2	-6.77
	Lingual gyrus	5611	18	R	-24.1	76.1	-8	-4.37
	Thalamus	2544		R	-16.8	18.5	-5.2	-3.82
	Precuneus	2364	7	L	30.4	47.4	44.4	-3.48
	Fusiform gyrus	1681	37	L	41.8	44.6	-17.8	-4.13
	Somatosensory association cortex	1501	5	R	-2.1	45.4	70.5	-4.01
	Precuneus	1445	7	R	-32.3	63.5	49.3	-3.47
	Cingulate gyrus	1441	23	R	-13.1	12.1	29.4	-3.00
	Anterior cingulate cortex	1251	24	R	-6	-27.7	-3.5	2.79
	Posterior cingulate cortex	995	29	R	-2	41	20.5	-3.44
	Middle temporal gyrus	938	39	L	57.3	63.7	9.5	3.68
	Primary motor cortex	904	6	R	-31.5	10.8	65.5	-3.55
	Anterior cingulate cortex	872	38	L	41.4	-19.9	-24.8	3.56
	Caudate	819		R	-0.6	-5.7	16.8	-2.78

Response delay	Superior frontal gyrus	2673	8	R	-13.5	-46.5	35.5	-3.144
	Superior temporal gyrus	1971	38	L	34.5	-19.5	-27.5	-4.197
	Postcentral gyrus	1944	6	L	55.5	7.5	11.5	-3.000
	Cerebellar tonsil	1377		R	-10.5	64.5	-39.5	-2.9166
	Insula	1296	13	R	-40.5	19.5	-0.5	-4.0758
	Insula	1242	13	L	49.5	-1.5	-24.5	-3.2954
	Precuneus	1134	7	R	-13.5	64.5	35.5	-3.1364
	Medial frontal gyrus	999	10	R	-10.5	-64.5	11.5	-3.6894
	Superior frontal gyrus	972	6	R	-10.5	-13.5	59.5	-2.9774
	Superior temporal gyrus	837	38	R	-43.5	-16.5	-27.5	-3.5607

**Figure 3:**
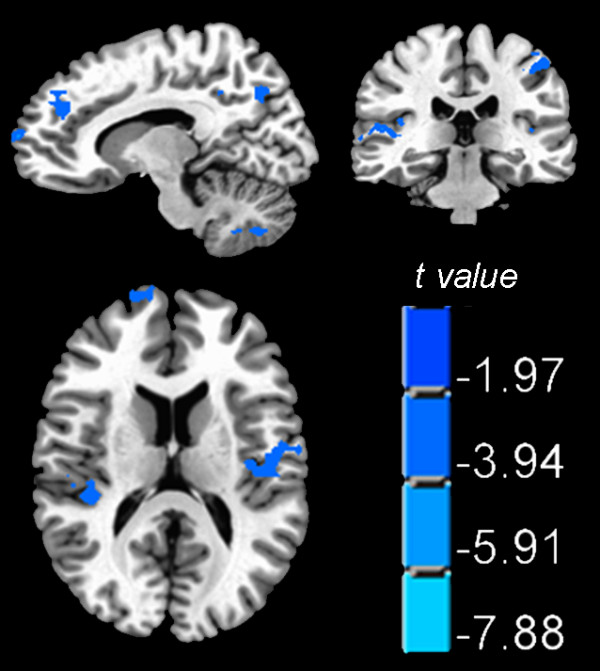
**Group comparison on the delay of hemodynamic response**. The HA group showed longer delay in multiple regions, particularly at the bilateral insula, which are shown on the axial plane.

Negative *t *values indicate decreased activation or longer response delay among the HA group. These clusters are also displayed in Figure 1 and Figure 3.

### Temporal patterns of hemodynamic response

**Figure 4:**
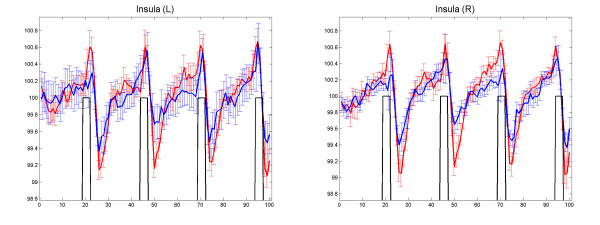
**The average BOLD signal extracted from the bilateral insula that showed significantly longer delay in Figure 3**. The BOLD signal of the HA group was slower to reach peak in response to the maximum inspiration task.

In terms of the temporal patterns of hemodynamic response, the HA group generally had longer delay than the SL control group, especially at the precuneus, the insula, the superior frontal and temporal gyrus, the somatosensory cortex (the postcentral gyrus) and the cerebellar tonsil (Figure 3, Table 2). Similar analysis for generating Figure 2 were conducted to generate Figure 4. As shown in Figure 4, the temporal delay in BOLD response was well manifested at bilateral insula.

### Correlations with pulmonary functions

**Figure 5:**
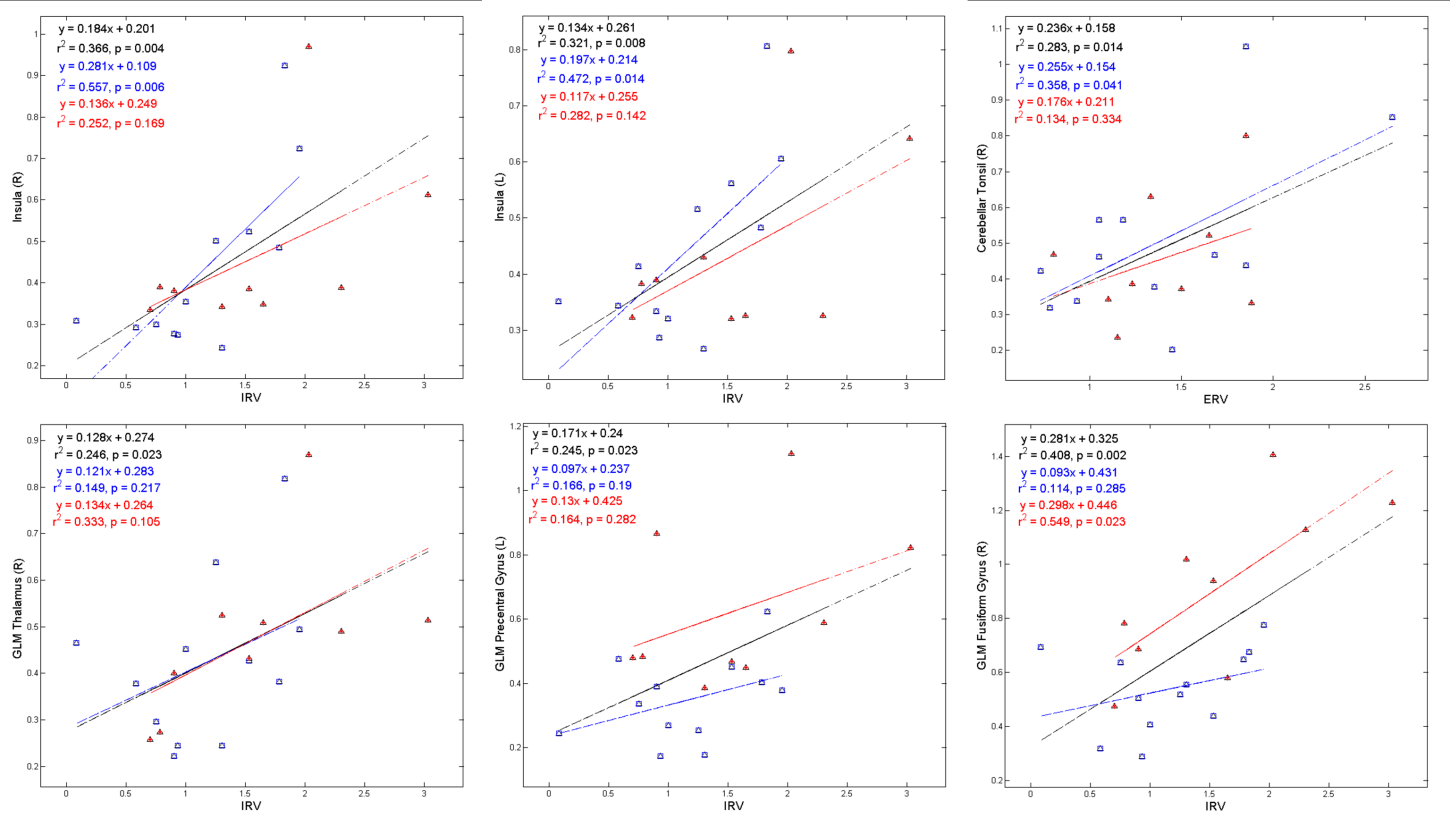
**Correlations between the pulmonary functions and the amount of BOLD signal change**. IRV: inspiratory reserve volume; ERV: expiratory reserve volume (ERV). Fitting lines, symbols and texts in blue color represent relevant information of the HA group, whereas fitting lines, symbols and texts in red color represent relevant information of the SL group, and those in black color represent corresponding information from the overall subject pool including both groups.

In order to study the correlations between the BOLD signal and pulmonary functions, for each individual subject, we first extracted the BOLD signal from the clusters that showed significant group differences (listed in Table 2), and then we calculated the standard deviation (SD) of the BOLD signal in each cluster and used these values (refereed to as BOLD-SD in this paper) to correlate with the scores from pulmonary function tests. The reason for using BOLD-SD is because during preprocessing, the BOLD signal of each voxel was already normalized with reference to its across time average, thus BOLD-SD reflects the amount of change in the signal, which reflects the degree of CVR. We found that among the subject pool containing both groups (the black lines in Figure 5), IRV (Inspiratory Reserve Volume) was significantly correlated with the BOLD-SD of the bilateral insula, the right thalamus, the left precentral gyrus and the right fusiform gyrus; ERV (Expiratory Reserve Volume) was significantly correlated with the BOLD-SD of the right cerebellar tonsil. The correlations showed different levels of significance in different groups. IRV was significantly correlated with the BOLD-SD of bilateral insula among the HA group, but not the SL group; ERV was significantly correlated with the BOLD-SD of the right cerebellar tonsil among the HA group, but not the SL group; on the other hand, IRV was significantly correlated with the BOLD-SD of the fusiform gyrus among the SL group, but not the HA group; IRV was significantly correlated with the BOLD-SD of the right thalamus and the precentral gyrus among the whole subject pool, but not among any single group.

## Discussion

This fMRI study investigated the CVR among native-born HA residents using a visually cued maximum inspiration paradigm with brief breath holding. CVR was significantly reduced among the HA group; several brain regions also showed longer delay in hemodynamic response; IRV as an important aspect of pulmonary function was significantly correlated with the amount of BOLD signal change at multiple brain regions.

During the task, influx of oxygen accompanied by maximum inspiration induces prompt rise of BOLD signal and the following expiration induces drop of the BOLD signal (Figure 2). In this process, cerebrovascualr properties can impact the BOLD signal; if CVR is greater, the change of the BOLD signal induced by the task shall be larger [14,15,21]. As shown in Figure 1~Figure 4, the HA group generally had smaller and slower BOLD response. This indicates generally decreased CVR among the HA group, which is consistent with the hypometabolism hypothesis in literature [3,4]. It was proposed that during HA adaptation, hypometabolism was developed as a mechanism to cope with the hypoxic stress. The hypometabolism observed on human brains has been compared with the hypometabolism in other species that are hypoxia tolerant such as the diving seal and the aquatic turtle. Such hypometabolism was also observed in our previous two fMRI studies involving gustatory stimulation [9] and cognitive performance [10].

Because a voluntary respiration task was adapted, clusters showing significant group differences are mainly located at brain regions typically related to various aspects of respiratory regulation [27]. The clusters showing significantly decreased CVR were located at the bilateral primary motor cortex, the right somatosensory association cortex, the right thalamus and the right caudate, the bilateral precuneus, the right cingulate gyrus and the right posterior cingulate cortex, as well as the left fusiform gyrus and the right lingual cortex (Figure 1). There was also a cluster showing increased CVR at the anterior cingulate cortex (Figure 1). Generally, these brain regions are either involved in respiration modulation, or are important for inhibitory control. The motor cortex controls respiratory muscles in human [25,28]. The thalamus has projections to the medulla in the brainstem which controls respiration cycle [29], and in particular, under hypoxia conditions the thalamus had inhibitory effects on the respiratory neurons of the medulla and reduces the frequency of respiration [30]. The caudate was previously reported to be associated with voluntary control of breathing [31]. The somatosensory regions were previously reported to be involved in inspiratory occlusion [32]. The cingulate gyrus is well known for inhibitory control [33]; in particular, the posterior cingulate cortex, known as a critical region of default mode [34,35], is also known to be important for motor inhibition [36]. The lingual and fusiform cortexes were activated because of the visual cues in our task; activation at the visual-related brain regions was also reported in a previous study that used visual cues for voluntary breath holding [15]. The significant group difference at the visual-related cortex indicates that the change in CVR was not restrained to respiratory-control related brain regions, but could have been a general effect across multiple brain regions as long as a consistent regional activation was elicited. The following could be a possible interpretation for the reduced CVR at the brain regions typically involved in respiratory control: under acute exposure to hypoxic stress, the cerebral cortex would modulate relevant mechanisms to enhance ventilation; however, with prolonged chronic HA exposure during which even enhanced ventilation could not increase oxygen supply to cortex, a hypometabolism mechanism could be developed to prevent hyperventilation, so as to reserve energy and to make optimal utilization of the limited oxygen. Such hypometabolism can be pervasive throughout the brain; in the current study, it is expressed in cortical regions typically involved in respiration control and motor inhibition and even vision because these regions were activated during the task thus the BOLD signals at these regions were most reliable (from a cross-subject perspective), thus group differences were demonstrated at these regions. On the other hand, there was increased activation at the anterior cingualte cortex, which is an area generally considered to be important for attention and cognitive control [37-43]; such increase can be interpreted as increased mental efforts among HA subjects to maintain attention while following task instructions; such increased attention was also previously observed during a working memory task among this population [11].

The HA group showed longer delay in hemodynamic response to the task, with clusters showing significant differences located at the superior and medial frontal gyrus, the superior temporal gyrus, the postcentral gyrus, the insula, and the precuneus. The reasons for a slower hemodynamic response are complicated. It could be related to the decreased CVR; or it could be related to the reduced neuron activity among these regions. A particularly interesting result is the delay of BOLD response at the bilateral insular cortices (Figure 4). It has been shown that the insular cortex plays an important role in respiratory modulation [26,44-47]. Our previous study found reduced amount of gray matter volumes at bilateral insula [8]. Such repeated findings of impairment at the insula points to its reduced functionality among the HA group. One plausible interpretation would be that such reduced functionality contributes to the reduced ventilation associated with long term HA adaptation, in contrast with the hyperventilation typically experienced by new comers. In future studies it would be interesting to investigate why prolonged HA exposure during early development leads to reduced volume and decreased functionality particularly at the insula.

Significant correlations were found between specific aspects of pulmonary function (IRV & ERV) and BOLD signal variation at specific brain regions. These correlations indicate the contribution of specific aspects of pulmonary function to CVR. In pulmonary function testing, the amount of air a person breathes in and out during quiet normal breathing is called the Tide Volume (VT). The additional amount a person could inhale is called the Inspiratory Reserve Volume (IRV). The additional amount a person could exhale is called the Expiratory Reserve Volume (ERV). It seems that IRV had a wide influence on BOLD signal across multiple brain regions, as shown in Figure 5, not only in cortices involved in respiratory modulation (e.g., the insula, thalamus, and the precentral cortex), but also other cortices such as the fusiform cortex which was activated merely due to the visual cues in the task. This indicates that IRV probably has a general impact on CVR, possibly because individual differences in IRV impacts the individual differences in the amount of intake oxygen, which further influences CVR. In particular, the correlation at bilateral insula might help to explain the longer delay of hemodynamic response among the HA group. The HA group indeed showed a larger IRV than the control group, while other aspects of pulmonary function maintained at a similar level. It could be possible that the it took a longer time for the oxygenation level of the HA group to reach peak, considering that the HA group had similar levels of respiration rate and haemoglobin concentration with the only observed difference at IRV; and this delay was eventually reflected in the delay of BOLD signal. Besides, the correlations between IRV and the BOLD-SD at the thalamus and the precentral gyrus could also be related to the involvement of these brain regions in respiratory modulation. The correlation between IRV and the BOLD-SD at the fusiform cortex, which was significant among the SL group but not the HA group, indicates that probably IRV among the HA group had a relatively more specific impact on respiration modulation, because respiratory modulation was very important for HA adaptation; whereas IRV could have a less specific (thus more general) impact on CVR among SL subjects. The correlation between ERV and the BOLD-SD at the cerebellar tonsil is harder to explain; since there was no significant group difference in ERV, and the function of cerebellar tonsil is not yet clear in literature; besides, in the scatter plot in Figure 2, distribution of data points in this sub-figure has a less definite pattern compared to other sub-figures, so we would take this result with caution considering our relatively small subject number. In summary, our results suggest the possible contributions of pulmonary functions in CVR; future investigation should probe into the exact physiological mechanisms of how pulmonary functions contribute to CVR.

We hope to point out that the current study purposefully controlled two factors that could have confounded group differences. Firstly, subjects of both groups were from the Han ethnic group so as to control the genetic factor between groups; in many previous studies in the HA literature, indigenous HA local residents were recruited with the control group being indigenous SL residents [3,48-50], such experiment design makes it difficult to dissociate the contribution of genetic and developmental effect; but in our study the genetic factor was controlled as much as possible by selecting subjects from the same ethnic group. Secondly, the HA subjects had been living at SL for at least two years at the time of experiment. It was reported that peripheral physiological parameters, especially hemoglobin concentration, adapt very quickly to hypoxia/normoxia changes in the scale of weeks [51]; and in our previous study with a larger sample size in which HA subjects had resided at SL for at least one year (in the current study the HA subjects had resided at SL for at least two years), the HA group indeed did not show significant difference on hemoglobin concentration (Table S2) [8]. Given the controlled genetic factor, and the physiological adaptation to normoxia associated with the long term SL adaptation of our subjects, it is more likely that the observed differences demonstrated the impact of prolonged HA exposure in early childhood on brain development.

There are limitations in the research technique employed in the current study. BOLD fMRI signal comes from multiple physiological factors [12,13,52], it does not provide direct measurement on cerebral blood flow, nor on metabolism, it cannot capture the possible differences in vascular structures either. In order to further explore the impact of HA exposure on the cerebral circulation system, future studies should attempt application of other MRI techniques, such as diffusion tensor imaging (DTI) [53] or time-of-flight MR angiography (MRA) [54,55], which can possibly reveal the fine scale differences in vascular structures; or arterial spin labelling (ASL) [56,57], which provides a more direct measurement of the cerebral blood flow, etc.

## Conclusions

We studied the CVR of native-raised HA residents with a visual-cue guided maximum inspiration paradigm. The HA group showed generally decreased CVR and delayed hemodynamic response, suggesting consistency with the hypometabolism hypothesis in literature. Such effect are mostly manifested among brain regions that are typically involved in respiration modulation. The hypometabolism of the HA group, particularly among the regions associated with respiration modulation, might have been an adaptive strategy during long term chronic hypoxia exposure. Such adaptation can reduce the hyperventilation that new comers typically experience, thus to preserve energy and make optimal use of the limited oxygen.

## Methods

### Subjects

Participants in the current study included 12 HA residents and 11 matched SL (< 400 m) residents. HA residents were born and raised at Qinghai-Tibetan Plateau for more than 20 years at the altitude of 2527-3958 m until they relocated at SL (< 400 m). The average ages of the HA and SL groups are 22.4 ± 1.7 and 24.8 ± 2.3 respectively (see Additional file 1). All subjects were from the Han ethnic group, and were living near SL at the time of the experiment. The groups were of similar age and had similar scores in the national examinations for college entrance. Self-report questionnaires indicated that none of the participants had a history of being diagnosed with neurological or psychiatric conditions, or any of the following problems: cigarette smoking, insomnia, dyspeptic disorders, recent physical injury, or a history of hospitalized surgery. Spirometry tests were conducted prior to the fMRI experiment with RSFJ-1000 (Chendu Risheng CO., LTD, Chendu, China). Due to logistic issues, 2 subjects in the SL group did not have their pulmonary function tested. 7 subjects in the HA group participated in a previous study [8], and 4 subjects in the SL group participated in the previous study [8]. The experimental protocol was approved by the Institute Review Board of the Institute of Psychology, Chinese Academy of Sciences. Written informed consent was obtained from each participant.

### MRI Data Acquisition

#### Scanning parameters

Anatomical and functional images were acquired on a GE 3.0 T Signa Excite Gemse MRI system (GE Medical, Milwaukee, WI, USA) at Huaxi Magnetic Resonance Research Center (West China Hospital, Chengdu, China). A 3D anatomical MRI was acquired from each subject using a T1-weighted MPRAGE sequence (TR/TE = 8.5 ms/3.4 ms, TI = 400 ms, FOV = 28 cm, flip angle = 12°), yielding 156 contiguous axial slices (1 mm thick) covering the whole brain. During the maximum inspiration task, T2*-weighted EPI sequence was applied with TR/TE = 2000/30 ms, FOV = 23 × 23 cm^2^, flip angle = 90°, 28 axial slices with 4 mm thickness and a 0.5 mm gap covering the whole brain were acquired. Foams were put in between the head and the coil to prevent from head motion during the scanning. Ear plugs and blankets were provided for the comfort of the subjects.

#### Visual cue guided maximum inspiration task protocol

Following a 42 second rest period, subjects were instructed to make a maximum inspiration within 2 seconds and then exhale slowly within 6 seconds. This was followed by another 42-second rest period. The procedure was repeated four times, yielding a total of 100 volumes (time points) (see Figure 2). Trial timing was cued by words presented on a black background in white color. The words included 'rest', 'get ready', 'inhale' and 'exhale'. Subjects viewed the cues through a mirror mounted on the head coil.

### Data Analysis

FMRI data were analyzed with AFNI (Analysis of Functional NeuroImages) software [58]. The following preprocessing procedures were conducted on the original BOLD signal: slice timing correction, motion correction, removal of linear drift and smoothing with a Gaussian filter of 6 mm FWHM. A motion profile of 6 parameters reflecting the amount of head motion was estimated for each subject from the motion correction step. None of the subjects' head motion in any direction exceeded the amount of one voxel, so no subject was excluded. The signals at each voxel of each subject were standardized by dividing the mean across time and multiplying by 100, reflecting how much the signal changed at each time point relative to the average across the whole time period.

To extract group differences in different aspects of CVR, the following analyses were conducted:

(1) In order to reveal the general pattern of CVR, we used GLM to fit the BOLD signal from each voxel with a stimulus function that specified the onset and ending time of the maximum inspiration events. The fitting was conducted with the 3dDeconvolve function in AFNI. Besides the stimuli reference, the motion profiles of 6 directions, which were obtained from the motion correction step during preprocessing, were also used as regressors in the 3dDeconvolve function. The maps of estimated coefficients (the beta values) were coregistered to the 3D anatomical image of each subject that had been normalized to the Talairach-Tournoux space [59]. Voxel-wise group statistics were conducted on the coregistered coefficient maps. The activation map of each group was identified with a one-sample t-test against 0. The group comparison was obtained with a two sample t-test, with threshold set at *p *< 0.05 (FWE corrected), cluster size > 800 mm^3 ^(Figure 1, Table 2).

(2) In order to explore whether the two groups have differences on temporal delay in the hemodynamic response to the task, we tentatively shifted the original stimulating sequence backward for 1, 2, 3, 4 and 5 seconds to explore which one has the best fit with the hemodynamic response in each voxel. Group differences were also explored with the independent two sample t-test (Figure 3, Table 2).

(3) We outlined the clusters that showed significant group differences as a result of the analysis in (1) and (2) to be used as masks, then extracted from the common masks the average of the preprocessed signal in the data of each individual subject, then averaged the extracted signal for each group. For demonstration purposes, we plotted the typical pairs of BOLD signal from these clusters in Figure 2 and 4. Furthermore, in order to investigate the relationships between pulmonary functions and BOLD response signals, we further calculated the SD of the extracted BOLD signals for each individual subject at each cluster, and calculated Pearson correlations between the BOLD-SD values and the scores in the pulmonary function tests (Figure 5).

## Authors' contributions

XY, JZ and XW designed the experiment, XY, JZ and GQ performed the experiment, XY analyzed the data and drafted the manuscript, all authors participated in critical revision of the manuscript and approved the final manuscript.

## Supplementary Material

Additional file 1 **Detailed information of subjects**. The supplementary document includes subjects' demographic information, as well as the demographic and physiological information of the subjects in our previous study [8] which showed no significant group differences on hematological measurements including hemoglobin levels (HGB) and circulating red blood cell count (RBC) etc. Click here for file
