# Supplementary material for: Cerebrovascular reactivity among native-raised high altitude residents: an fMRI study
Source: BMC Neurosci. 2011 Sep 26;12:94. doi: 10.1186/1471-2202-12-94 (PMC3213017; doi:10.1186/1471-2202-12-94)
Supplement: Additional file 1 — Detailed information of subjects. The supplementary document includes subjects' demographic information, as well as the demographic and physiological information of the subjects in our previous study [8] which showed no significant group differences on hematological measurements including hemoglobin levels (HGB) and circulating red blood cell count (RBC) etc. [file 1471-2202-12-94-S1.DOC]

**Supplementary material for**

**Cerebrovascular reactivity among native-raised high altitude residents: an fMRI study**

Xiaodan Yan^1,3,4^, Jiaxing Zhang^1,2^, Qiyong Gong^5^, Xuchu Weng^1^

^1^Laboratory for Higher Brain Function, Institute of Psychology, Chinese Academy of Sciences, Beijing, China.

^2^Department of Physiology, Medical College of Xiamen University, Xiamen, China.

^3^Cognitive Science Department, Rensselaer Polytechnic Institute, Troy, NY, USA

^4^Center for Neural Science, New York University, New York, NY, USA.

^5^Huaxi Magnetic Resonance Research Center, West China Hospital, Sichuan University, Chengdu, China.

Email addresses:

XY: xiaodan.yan@yahoo.com

JZ: xzhangj@163.com

QG: qiyonggong@hmrrc.org.cn

XW: wengxc@psych.ac.cn

This supplementary document contains 3 tables.

This supplementary document demonstrates the demographic information of the subjects in the current study (Table S1), as well as the demographic (Table S2) and physiological information (Table S3) of the subjects in our previous study[1]. The purpose of the pervious study was to comprehensively explore the impact of HA residence on brain structure and function; whereas the current study was to specifically study the cerebrovascaular reactivity. The current study was conducted about 1 year after the previous study.

In the previous study, we examined many aspects of peripheral physiology (Table S3); blood samples were drawn and analyzed with hematology analyzer Sysmex XE-2100 (TOA Medical Electronics, Kobe, Japan); pulmonary function was examined with RSFJ-1000 (Chendu Risheng CO., LTD, Chendu, China). Because HA subjects had been living at SL for more than one year at the time of testing , they did not show significant difference with their SL peers on hematological measurements including hemoglobin levels (HGB) and circulating red blood cell count (RBC), because these parameters adapt very quickly to hypoxia/normoxia changes [2]. Male subjects showed difference on inspiratory reserve volume (IRV) of lung function, which was also manifested among the male subjects in the current study (Table 1).

Subjects in the current study have similar demographic characteristics with those in the previous study, except for that they had been residing at SL for a longer time (at least 2 years, with average of about 3 years). Because literature[2] and our own previous study[1] have demonstrated quick and well adaptation of hematological parameters, we did not do hematological examination in the current study, but focused our attention on pulmonary functions (Table 1 & Figure 5 in the main text).

**Reference**

1. Yan X, Zhang J, Shi J, Gong Q, Weng X: **Cerebral and functional adaptation with chronic hypoxia exposure: A multi-modal MRI study**. *Brain research* 2010, **1348**:21-29.

2. Neubauer JA: **Physiological and genomic consequences of intermittent hypoxia: Invited Review: Physiological and pathophysiological responses to intermittent hypoxia**. *J Appl Physiol* 2001, **90**(4):1593-1599.

**1. Table S1.** Demographic information of the subjects in the current study.

|  | HA group | SL group |
| --- | --- | --- |
| Number of subjects  Ethnic origin  Ages [Mean (SD)]  Altitudes of residence [Mean (SD)]  Time of relocation at SL  Education level [Mean (SD)] | 12 (male:6, female: 6)  Han  22.4 (1.7) yrs  3012.7 (497.8) m  2.8 (1.6) yrs  14.5 (0.9) yrs | 11 (male:5, female:6)  Han  24.8 (2.3) yrs  < 400 m  -  15.6 (1.4) yrs |

**2. Table S2.** Demographic information of the subjects in our previous study[1].

|  | HA group | SL group |
| --- | --- | --- |
| Number of subjects  Ethnic Origin  Ages [Mean (SD)]  Altitudes of residence [Mean (SD)]  Generations of residence at HA  Time of relocation at SL  Education level [Mean (SD)]  Education of parents [Mean (SD)] | 28 (male:12, female:16)  Han  20.4 (1.4) yrs  2982.8 (478.7) m  2 (28%), 3 (43%), > 3 (29%) yrs  1 (18%), 2 (32%), > 3 (50%) yrs  13.3 (0.8) yrs  8.8 (3.4) yrs | 28 (male:12, female:16)  Han  20.9 (1.5) yrs  < 400 m  -  -  13.4 (0.6) yrs  9.2 (1.8) yrs |

**3. Table S3**. Physiological characteristics of the subjects in our previous study [1].

|  | HA | SL |
| --- | --- | --- |
|  | Males Females  (12) (16) | Males Females  (12) (16) |
| **Body Height (cm)**  **Body Weight (kg)**  **Hematological measurements**  HGB (g/L)  RBC (×10^12^ )  **Blood pressure** (Kpa)  (Resting state/Maximal inhaling state)  Systolic pressure  Diastolic pressure  **Pulse Rate** (times/min)  (Resting state/Maximal inhaling state)  **Pulmonary Function testing**  VC (liters)  IRV (liters)  TV (liters)  IC (liters)  MVV (real /estimated %)  RR (times /min)  FVC (real /estimated %)  FEV1 (real /estimated %)  %FEV1(real/estimated %)  MMEF(real/estimated%)  PEFR (real /estimated %) | 176.7 ± 4.6** 163.3 ± 5.4*  59.3 ± 6.0 50.5 ± 5.3  145.60 ± 9.45 136.60 ± 7.27  5.02 ± 0.61 4.45 ± 0.28  119.8 ± 10.3 / 116.5 ± 17.1 110.3 ± 6.6 / 112.9 ± 5.1  75.2 ± 5.2 / 75.5 ± 12 67.0 ± 9.6/ 66.3 ± 8.6  70.6 ± 11.6 / 73.3 ± 8.5 76.9 ± 6.8 / 73.6 ± 8.8  2.97 ± 1.64 3.19 ± 0.51  1.12 ± 0.69* 1.20 ± 0.38  0.86 ± 0.41 0.73 ± 0.19  1.59 ± 1.04 1.88 ± 0.38  143.64 ± 29.92 111.98 ± 6.31  19.61 ± 7.41 19.58 ± 3.31  95.23 ± 31.47 96.64 ± 11.13  101.07 ± 26.96 102.53 ± 6.54  110.68 ± 7.67 112.56 ± 8.70  87.98 ± 23.42 88.72 ± 7.02  104.4 ± 16.22 86.43 ± 11.58 | 171.2 ± 5.5 159.1 ± 5.4  57.0 ± 5.9 50.2 ± 4.6  149.0 ± 3.1 132 ± 2.1  5.08 ± 0.7 4.33 ± 0.4  115.3 ± 11.4 / 111.6 ± 9.4 108.5 ± 7.4 / 107.8 ± 8.1  76.6 ± 9.6 / 71.0 ± 8.1 67.3 ± 4.7 / 65.4 ± 5.3  77.4 ± 6.6 / 74.8 ± 8.9 74.9 ±4 .8 / 72.1 ± 10.6  3.72 ± 0.30 2.78 ± 0.34  2.06 ± 0.66 1.09 ± 0.34  0.68 ± 0.38 0.64 ± 0.30  2.25 ± 0.50 1.56 ± 0.34  141.29 ± 29.95 111.34 ± 6.90  16.58 ± 5.12 18.12 ± 6.45  92.86 ± 5.72 100.21 ± 14.88  92.00 ± 8.81 104.10 ± 7.29  101.95 ± 7.16 117.26 ± 8.43  81.36 ± 25.68 89.95 ± 14.87  87.64 ± 17.68 93.52 ± 9.97 |

Abbreviations: RBC: (count of) red blood cells; HGB: hemoglobin; VC, vital capacity; TV, tide volume; IRV, inspiratory reserve volume; IC, inspiratory capacity; MVV, maximum voluntary ventilation; RR, respirotary rate; FVC, forced vital capacity; FEV1, forced expired volume in one second; MMEF, mean mid-expiratory flow; PEFR, peak expiratory flow rate. Data are presented in the form of Mean (SD). **p* < 0.05, ***p* < 0.01.

Note: The HA group were significantly taller than the SL group, we did not see any neurological or cognitive implication of this effect, but at least this shows that HA group did not suffer from malnutrition.
